# Smiles That Matter: A Pilot Study on the Oral Health and Quality of Life of 8-10-Year-Olds Using the Child Perceptions Questionnaire 8-10 in the Rayagada District of Odisha, India

**DOI:** 10.7759/cureus.91094

**Published:** 2025-08-27

**Authors:** Payal Dash, Anandamoy Bagchi, Ipseeta Menon, Gunjan Kumar

**Affiliations:** 1 Department of Public Health Dentistry, Kalinga Institute of Dental Sciences (KIDS) Kalinga Institute of Industrial Technology (KIIT) Deemed to be University, Bhubaneswar, IND; 2 Department of Pediatrics and Preventive Dentistry, Kalinga Institute of Dental Sciences (KIDS) Kalinga Institute of Industrial Technology (KIIT) Deemed to be University, Bhubaneswar, IND

**Keywords:** child, dental caries, india, oral health, oral health surveys, quality of life

## Abstract

Background

Oral health-related quality of life is a key indicator of well-being among children, particularly in rural and underserved populations. This pilot study aimed to assess dental caries experience and its association with oral health-related quality of life using the Child Perceptions Questionnaire (CPQ) among 8-10-year-old schoolchildren in Derigram Panchayat, Rayagada district, Odisha.

Methodology

A cross-sectional study was conducted among 112 children selected using stratified random sampling. Dental caries were evaluated using the World Health Organization criteria (2013), and oral health-related quality of life was examined using the CPQ₈₋₁₀, which includes the following four domains: Oral Symptoms, Functional Limitations, Emotional Well-being, and Social Well-being. Data were analyzed using descriptive statistics, analysis of variance, Kruskal-Wallis test, and Spearman’s rank correlation test. A probability value of less than 0.05 was considered statistically significant.

Results

The mean number of decayed, missing, and filled teeth in the primary dentition was the highest among nine-year-olds (3.43 ± 2.05), with a total sample mean of 3.07 ± 1.88. Statistically significant differences in caries scores were observed across age groups for the number of decayed teeth in the primary dentition (p = 0.043); the overall decayed, missing, and filled teeth score in the primary dentition (p = 0.036); and the number of decayed teeth in the permanent dentition (p = 0.039). Spearman’s rank correlation test showed significant positive associations between the overall decayed, missing, and filled teeth score in the primary dentition (correlation coefficient = 0.40), the number of decayed teeth in the primary dentition (correlation coefficient = 0.36), and the overall decayed, missing, and filled teeth score in the permanent dentition (correlation coefficient = 0.38) with the total CPQ scores (all p < 0.01), particularly within the Social Well-being domain.

Conclusions

The study highlights a moderate caries burden among rural schoolchildren and demonstrates a significant negative impact of untreated dental caries on children’s quality of life. Findings emphasize the need for early intervention and school-based preventive programs, especially in tribal and underserved regions. Larger studies are needed to validate these preliminary observations and inform targeted oral health policies. Furthermore, the application of the CPQ₈₋₁₀ in this rural Indian context provides novel insights, underscoring the importance of culturally relevant, child-centered quality of life measures in oral health research.

## Introduction

The concept of health has evolved beyond the traditional biomedical model, which defined health merely as the absence of disease. Modern perspectives now embrace a more holistic understanding, one that includes physical, psychological, and social well-being. In this context, oral health assessment requires both objective clinical measures (such as dental caries indices determined by professionals) and subjective self-reported measures (such as oral health-related quality of life (OHRQoL)), ensuring that evaluations reflect not only clinical outcomes but also the lived experiences of individuals [1,2].

Oral health is a domain where the integration of clinical and self-reported outcomes is particularly important. Oral conditions, such as dental caries, malocclusion, and gingivitis, can affect fundamental activities such as eating, speaking, and interacting socially, especially in children. These disruptions can impair school performance, reduce self-esteem, and influence long-term health behaviours. Thus, the OHRQoL concept has gained prominence in pediatric and public health dentistry [3,4].

To assess OHRQoL in children, researchers and clinicians require developmentally appropriate instruments, psychometrically sound and sensitive to the specific challenges faced by younger populations. Several validated instruments currently exist to measure children’s OHRQoL [5]. Among these tools, the Child Perceptions Questionnaire for 8-10-year-olds (CPQ₈₋₁₀) has emerged as a widely validated instrument in Western populations [5,6]; however, to date, no formal validation studies have been reported in the Indian context. In contrast, the CPQ₁₁₋₁₄ has been validated in Indian populations, supporting the cultural applicability of CPQ in this region [7,8]. The CPQ₈₋₁₀ is specifically designed to capture children’s perceptions of how oral health affects their everyday lives across the following four domains: Oral Symptoms, Functional Limitations, Emotional Well-being, and Social Well-being. Unlike tools designed for adolescents or adults, the CPQ₈₋₁₀ accounts for the cognitive and emotional developmental stage of younger children, offering reliable insights into their subjective experiences with oral health [9].

While the CPQ₈₋₁₀ has been validated across several cultural settings, its use in underserved, rural, or tribal regions remains limited [10-12]. Rayagada district in southern Odisha, a predominantly tribal area, is marked by structural and behavioral barriers to oral health, including inadequate access to dental care, poor hygiene practices, and limited parental awareness. The district also records one of the lowest literacy rates in India (49.8%) and a Human Development Index of 0.18, far below the national average of 0.645. More than half of its residents belong to Scheduled Tribes, and the majority live in rural, underserved settings [13]. These socioeconomic vulnerabilities may profoundly influence both oral health and overall quality of life. Although studies from other settings consistently demonstrate the negative impact of dental caries and poor oral hygiene on children’s OHRQoL, such evidence remains scarce for this particularly vulnerable population [14,15].

Based on this background, the current study was designed to assess the oral health condition and OHRQoL of 8-10-year-old schoolchildren in the Rayagada district.

## Materials and methods

Study design and setting

This cross-sectional study included children aged 8-10 years in Derigaon Panchayat, Rayagada district, Odisha. The study was approved by the Ethics Committee of Kalinga Institute of Medical Sciences, Bhubaneswar (reference number: KIIT/KIMS/IEC/1912/2024). Written informed consent was obtained from parents/guardians of all participating children, and verbal assent was taken from the children before the oral examination. Local permission to conduct the study was obtained from the village Sarpanch, and community mobilization was facilitated with the support of the Anganwadi worker. Participation was voluntary, and confidentiality was maintained throughout.

Study population, sampling method, and sample size

**Figure 1:**
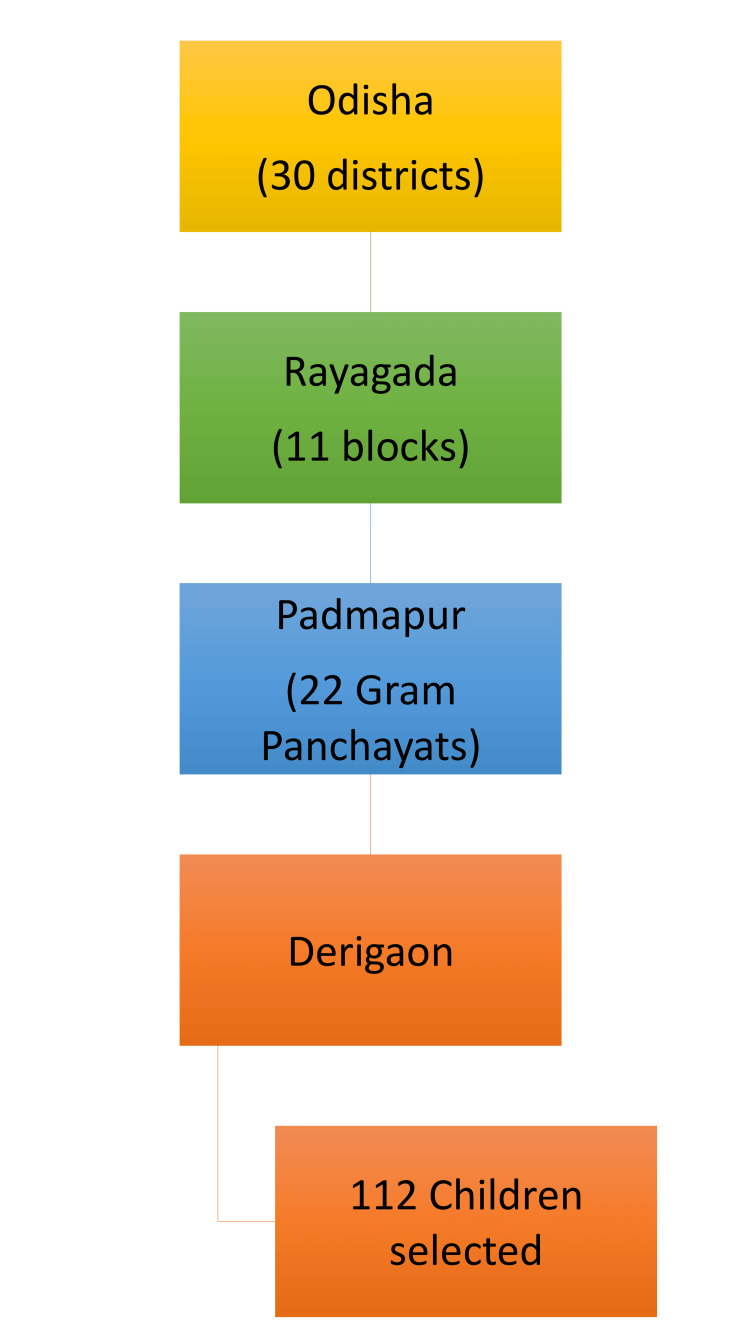
The sampling method employed in the study.

As per the 2018-2019 Unified District Information System for Education report, Rayagada district had a total child population in the 6-14-year age group of approximately 208,215, indicating the school-going population in that range [16]. The target population consisted of healthy school-going children aged 8-10 years. Although the predicted minimum sample size for a full-scale cross-sectional investigation was 288, based on a 25% expected prevalence of poor OHRQoL, a 5% margin of error, and a 95% confidence level, a smaller sample size was determined to be adequate for this pilot study. As no prior data were available on the prevalence of poor OHRQoL in this population, an expected prevalence of 25% was assumed for the sample size calculation. Considering the exploratory nature of the study and logistical feasibility, a total of 112 children were included using multistaged stratified random sampling (Figure 1).

Inclusion and exclusion criteria

The study included children aged 8-10 years who were present on the day of the examination and participated after providing informed assent, along with written parental consent. Children with special healthcare needs and those whose parents did not provide consent were excluded from the study.

Data collection tools

Oral health status was assessed using the modified WHO Oral Health Assessment Form (2013), focusing on decayed, missing, and filled teeth (DMFT/def indices), gingival status (healthy, bleeding, calculus), and oral hygiene practices (materials used, type of cleaning, method, and frequency of brushing [17]. Box numbers 37,38,39, and 40 in the other data section were used to code materials used for oral hygiene practices. Examinations were conducted under natural light using mouth mirrors and CPI probes, adhering to WHO examination protocols.

The validated short-form CPQ₈₋₁₀ was used to evaluate children’s quality of life connected to dental health. The CPQ₈₋₁₀ questionnaire was adapted from Ju et al., which is available under an open-access license [6]. The questionnaire was translated into Odia using a forward-backward translation method. This version includes eight items divided into the following four conceptual domains: Oral Symptoms (pain, food stuck or caught), Functional Limitations (difficulty biting or chewing firm food, taking longer to eat a meal), Emotional Well-being (being upset due to oral problems, irritability or frustration), and Social Well-being. Each item was rated on a five-point Likert scale (1 = never, 5 = very often). Responses were recoded from 1-5 to 0-4 for analytical purposes. The overall CPQ₈₋₁₀ score varied from 0 to 16, with higher values suggesting lower OHRQoL.

Data collection procedure

Data collection for the present study was conducted using a combination of digital and traditional methods to ensure completeness and accuracy. The semi-structured questionnaire, which included the CPQ₈₋₁₀ items and sociodemographic details (Appendices), was digitized and administered through KoBoToolbox (https://ee.kobotoolbox.org/x/QaLHM477), a secure, open-source platform designed for field data collection in challenging environments. To avoid any diagnostic variability among the study participants, a single examiner was trained, and the instrument was calibrated in the Department of Public Health Dentistry. Data collection was performed by the investigator herself using mobile devices to collect responses in real-time, ensuring immediate data capture, minimal data entry errors, and enhanced efficiency in data management. Intra-examiner reliability was tested on a subset of children (n = 10), with duplicate examinations performed one week apart, yielding a Cohen’s kappa of 0.86, indicating good reliability.

In parallel, clinical oral health status was assessed following the WHO Oral Health Assessment Form (2013) guidelines. Type three clinical examination was performed as per American Dental Association specifications using plain mouth mirrors and CPI probes under adequate natural illumination. Subjects were made to sit on a chair in an upright position. A total of 25-30 subjects were examined every day. The clinical findings were recorded on hard copies of the WHO Assessment Form, which were later manually verified and digitized for analysis. This dual approach allowed cross-validation between digital entries and paper-based clinical observations, ensuring data integrity and reducing the risk of missing or incorrect information.

Statistical analysis

Data were entered into Microsoft Excel (Microsoft Corp., Redmond, WA, USA) and analyzed using SPSS Statistics version 27 (IBM Corp., Armonk, NY, USA). Descriptive statistics, including mean, standard deviation, frequency, and percentages, were used to summarize demographic details, caries indices (dmft/DMFT, dt, mt, ft), and CPQ₈₋₁₀ domain scores. Normality of the data was assessed using the Shapiro-Wilk test. As the data did not follow a normal distribution for several variables, non-parametric tests were used where appropriate. Spearman’s rank correlation was used to assess the relationship between caries experience (dmft/DMFT and subcomponents) and CPQ₈₋₁₀ domain scores (Oral Symptoms, Functional Limitations, Emotional Well-being, Social Well-being). The Kruskal-Wallis H test was employed to compare CPQ scores across categories of gingival health (healthy, bleeding, calculus). All statistical tests were two-tailed with a significance level set at p-values <0.05. No formal correction for multiple comparisons was applied, as this was an exploratory pilot study intended to generate preliminary findings rather than definitive inferences.

## Results

The age and gender distribution of the study participants (n = 112) showed that the majority were male children, comprising 67.0% of the sample. Most children (87.5%) used toothbrushes, while a few used *Karanja* twigs (*Millettia pinnata*) (10.7%) or fingers (1.8%). With respect to brushing frequency, the majority of children (85.7%) brushed once per day, and only 14.3% brushed twice daily, falling short of the recommended oral hygiene guidelines. This low prevalence of twice-daily brushing further supports the need for oral health promotion and school-based behavioral interventions (Table 1).

**Table 1:** Distribution of participants by age, gender, and oral hygiene practices.

Age (years)	Female, n (%) n = 37	Male, n (%) n = 75	Total, n (%) n = 112
8	8 (7.1%)	35 (31.3%)	43 (38.4%)
9	18 (16.1%)	31 (27.7%)	49 (43.8%)
10	11 (9.8%)	9 (8.0%)	20 (17.9%)
Type of cleaning			
Finger	1 (0.9%)	1 (0.9%)	2 (1.8%)
Twigs	10 (8.9%)	2 (1.8%)	12 (10.7%)
Toothbrush	64 (57.1%)	34 (30.4%)	98 (87.5%)
Material used			
Herbal toothpaste	31 (27.7%)	21 (18.8%)	52 (46.4%)
Fluoridated toothpaste	43 (38.4%)	15 (13.4%)	58 (51.8%)
Toothpowder	1 (0.9%)	1 (0.9%)	2 (1.8%)
Method of cleaning			
Horizontal	31 (27.7%)	21 (18.8%)	52 (46.4%)
Vertical	41 (36.6%)	16 (14.3%)	57 (50.9%)
Circular	3 (2.7%)	0 (0.0%)	3 (2.7%)
Frequency of brushing			
Once per day	64 (57.1%)	32 (28.6%)	96 (85.7%)
Twice per day	11 (9.8%)	5 (4.5%)	16 (14.3%)
Total	37 (33.0%)	75 (67.0%)	112 (100.0%)

The item-wise CPQ₈₋₁₀ analysis revealed a high burden of oral health problems. Most children (73.2%) frequently experienced dental pain, while many reported food sticking in teeth (44.6% once, 42.9% sometimes) and difficulty chewing firm foods (52.7% once, 34.8% sometimes), indicating compromised oral hygiene and early functional limitations (Table 2).

**Table 2:** CPQ8–10 item-wise responses. CPQ₈₋₁₀: Child Perceptions Questionnaire for 8-10-year-olds

CPQ item	Never, n (%)	Once/Once or twice, n (%)	Sometimes, n (%)	Very often, n (%)
Pain in teeth during the last 3 months	–	13 (11.6%)	17 (15.2%)	82 (73.2%)
Food stuck in teeth during the last 3 months	–	50 (44.6%)	48 (42.9%)	14 (12.5%)
Difficulty in biting/chewing firm foods	7 (6.3%)	59 (52.7%)	39 (34.8%)	7 (6.3%)
Taking longer than others to eat a meal	14 (12.5%)	43 (38.4%)	48 (42.9%)	7 (6.3%)
Feeling upset due to teeth, lips, mouth, or jaws	–	52 (46.4%)	46 (41.1%)	14 (12.5%)
Feeling frustrated because of teeth or mouth	6 (5.4%)	37 (33.0%)	55 (49.1%)	14 (12.5%)
Missed school due to pain or dental appointments	20 (17.9%)	23 (20.5%)	48 (42.9%)	21 (18.8%)
Avoided talking to other children due to teeth or mouth problems	–	55 (49.1%)	50 (44.6%)	7 (6.3%)

Additionally, over 42.9% of children reported sometimes taking longer to finish meals, while 38.4% reported this happening once, possibly indicating discomfort while chewing. Regarding emotional well-being, 46.4% of children reported feeling upset once or twice, and 41.1% reported sometimes being upset due to problems in their teeth, lips, or jaws. Social functioning was also affected; 42.9% had missed school sometimes, and 18.8% missed it often due to pain or dental appointments. Furthermore, 49.1% of children did not want to talk to other children once, and 44.6% experienced this sometimes, indicating a negative psychosocial impact of oral health issues.

The mean scores across the four CPQ₈₋₁₀ domains indicated variation in the self-reported impact of oral conditions on children’s daily lives. The highest mean score was observed in the Oral Symptoms domain (6.29 ± 1.21), followed by Emotional Well-being (5.35 ± 1.21), Social Well-being (5.20 ± 1.46), and Functional Limitations (4.84 ± 1.18) (Table 3).

**Table 3:** Domain-wise CPQ8–10 scores among study participants (n = 112). Descriptive statistics of domain-wise CPQ₈₋₁₀ scores among 8–10-year-old schoolchildren in Rayagada district (n = 112). Values are presented as mean ± SD. CPQ₈₋₁₀: Child Perceptions Questionnaire for 8-10-year-olds; CI: confidence interval; SD: standard deviation

CPQ domain	Mean ± SD	95% CI for mean
Oral Symptoms	6.29 ± 1.21	6.06–6.53
Functional Limitations	4.84 ± 1.18	4.62–5.06
Emotional Well-being	5.35 ± 1.21	5.12–5.58
Social Well-being	5.20 ± 1.46	4.93–5.47

An age-wise comparison of dental caries indices revealed statistically significant differences across age groups for dt, dmft, and DT scores. The mean dt score was highest among nine-year-olds (2.96 ± 2.19), followed by eight-year-olds (2.56 ± 1.86), and lowest in 10-year-olds (1.65 ± 1.35), with a statistically significant difference (F = 3.245, p = 0.043). Similarly, dmft scores also differed significantly among age groups (F = 3.423, p = 0.036), with nine-year-olds again showing the highest mean score (3.43 ± 2.05). For the permanent dentition, DT scores were highest among eight-year-olds (1.16 ± 1.51) and decreased with age, showing a significant difference (F = 3.352, p = 0.039) (Table 4).

**Table 4:** Mean (±SD) scores of caries indices (primary and permanent dentition) across different age groups. Statistical test: one-way analysis of variance. *: p < 0.05. CPQ₈₋₁₀: Child Perceptions Questionnaire for 8-10-year-olds; CI: confidence interval; SD: standard deviation

Age group	Dt, mean ± SD (95% CI)	Mt, mean ± SD (95% CI)	Dmft, mean ± SD (95% CI)	DT, mean ± SD (95% CI)	MT, mean ± SD (95% CI)	DMFT, mean ± SD (95% CI)
8 years	2.56 ± 1.86 (1.99–3.13)	0.53 ± 0.50 (0.38–0.69)	3.09 ± 1.81 (2.54–3.65)	1.16 ± 1.51 (0.70–1.63)	0.09 ± 0.29 (0.00–0.18)	1.26 ± 1.68 (0.74–1.77)
9 years	2.96 ± 2.19 (2.33–3.59)	0.47 ± 0.54 (0.31–0.63)	3.43 ± 2.05 (2.84–4.02)	0.82 ± 0.83 (0.58–1.06)	0.00 ± 0.00 (–)	0.82 ± 0.83 (0.58–1.06)
10 years	1.65 ± 1.35 (1.02–2.28)	0.50 ± 0.51 (0.26–0.74)	2.15 ± 1.27 (1.56–2.74)	0.40 ± 0.50 (0.16–0.64)	0.10 ± 0.31 (–0.04–0.24)	0.50 ± 0.69 (0.18–0.82)
Total	2.57 ± 1.98 (2.20–2.94)	0.50 ± 0.52 (0.40–0.60)	3.07 ± 1.88 (2.72–3.42)	0.88 ± 1.13 (0.66–1.09)	0.05 ± 0.23 (0.01–0.10)	0.93 ± 1.24 (0.70–1.16)
F-value	3.245	0.179	3.423	3.352	2.517	3.020
P-value	0.043*	0.836	0.036*	0.039*	0.085	0.053

No statistically significant differences were observed in mt, MT, DMFT, or FT scores across the age groups (p > 0.05). While MT showed a borderline non-significant trend (p = 0.085), the ft and FT components remained zero across all age groups, reflecting the absence of filled teeth in both primary and permanent dentition. These findings suggest that untreated dental caries in the primary dentition peaks around age nine, while involvement of the permanent dentition remains limited but begins as early as age eight (Table 4).

Spearman’s correlation analysis revealed that among the various dental caries indices, only dt (decayed teeth in the primary dentition) and dmft (total caries experience in the primary dentition) showed statistically significant positive correlations with the Social Well-being domain of the CPQ₈₋₁₀. Specifically, dt was moderately correlated with Social Well-being (ρ = 0.224, p = 0.018), and dmft showed a slightly stronger correlation (ρ = 0.257, p = 0.006) (Table 5).

**Table 5:** Spearman’s correlation coefficients between caries indices and CPQ8–10 domains. Statistical test: Spearman’s rank correlation coefficient test. *: p < 0.05; **: p < 0.01. CPQ₈₋₁₀: Child Perceptions Questionnaire for 8-10-year-olds

Caries index	Oral Symptoms	Functional Limitations	Emotional Well-being	Social Well-being
dt	–0.077	0.132	–0.028	0.224*
mt	0.075	–0.101	0.102	0.054
dmft	–0.060	0.095	–0.008	0.257**
DT	0.143	0.148	0.132	0.063
MT	0.135	0.170	0.030	0.043
DMFT	0.163	0.163	0.137	0.068

No significant correlations were found between caries indices and the Oral Symptoms, Functional Limitations, or Emotional Well-being domains (p > 0.05 for all), indicating that while dental caries may not heavily impact children’s self-reported symptoms or emotions, it does have a measurable effect on their social confidence and participation. Additionally, indices related to the permanent dentition (DT, MT, DMFT) showed weak, non-significant associations with all CPQ domains, possibly due to the younger age of participants, where the involvement of permanent teeth is minimal.

A Kruskal-Wallis test was conducted to assess whether gingival health status (categorized as healthy, bleeding, or calculus) had a significant effect on the four CPQ₈₋₁₀ domains: Oral Symptoms, Functional Limitations, Emotional Well-being, and Social Well-being. The results showed no statistically significant differences among the groups for any of the domains (p > 0.05 for all). Although children with healthy gingiva showed a slightly higher mean rank for oral symptoms, and those with calculus showed slightly higher mean rank for functional limitations, these differences were not statistically meaningful. This suggests that gingival health status was not significantly associated with self-perceived OHRQoL among the children in this study (Table 6).

**Table 6:** Association between gingival health status and CPQ8–10 domains. Statistical test: Kruskal-Wallis H test. CPQ₈₋₁₀: Child Perceptions Questionnaire for 8-10-year-olds

CPQ domain	Gingival health status	N	Mean rank	χ² (df = 2)	*P*-value
Oral Symptoms	Healthy	24	66.69	3.474	0.176
	Bleeding	68	52.99		
	Calculus	20	56.20		
Functional Limitations	Healthy	24	55.56	0.881	0.644
	Bleeding	68	55.13		
	Calculus	20	62.30		
Emotional Well-being	Healthy	24	48.10	2.191	0.334
	Bleeding	68	59.01		
	Calculus	20	58.05		
Social Well-being	Healthy	24	57.98	0.076	0.963
	Bleeding	68	56.28		
	Calculus	20	55.48		

## Discussion

This pilot study assessed dental caries experience and gingival status and their impact on OHRQoL among 8-10-year-old schoolchildren in Derigaon Panchayat of Rayagada district, Odisha, using the validated CPQ₈₋₁₀. The study aimed to bridge the existing gap in the literature regarding subjective oral health perceptions among tribal and rural children, where service coverage and awareness remain limited.

The mean dmft scores were notably highest among nine-year-olds (3.43 ± 2.05), followed by eight-year-olds (3.09 ± 1.81), and lowest in 10-year-olds (2.15 ± 1.27). These results align with studies conducted in India, where similar trends were observed-caries peaked between ages seven and nine, followed by a gradual decline due to exfoliation of primary teeth [18,19]. Another study also reported that 43% had zero decayed, missing, and filled primary and permanent tooth surface (dmfs/DMFS), while 170 (23.9%) had more than five dmfs/DMFS [20]. These results are consistent with reports from other underserved populations, where caries peaks in the early school years and declines with exfoliation of primary teeth. Studies among Australian Aboriginal and Canadian First Nations have shown similarly high caries experience in primary dentition, reinforcing that children in socioeconomically disadvantaged or tribal communities worldwide share comparable risk patterns [21,22].

The complete absence of filled teeth in this study highlights a significant unmet treatment need. This finding mirrors patterns seen in rural populations across India and Southeast Asia, where extractions and neglect dominate due to limited dental workforce and cost-related barriers [23,24]. Similar findings were also found in a study done by Dash et al. [25].

Importantly, the present study found statistically significant positive correlations between caries indices, particularly dmft (ρ = 0.40, p = 0.001) and dt (ρ = 0.36, p = 0.002), and total CPQ scores, indicating that higher untreated caries were associated with poorer self-perceived quality of life. Among the CPQ domains, Social Well-being demonstrated the strongest correlation with dt and dmft. This suggests that children with visible caries may experience embarrassment, teasing, and reduced willingness to participate in group activities. The clinical consequences of untreated dental caries showed significant associations with the total CPQ₈₋₁₀ score, as well as with the Oral Symptoms and Functional Limitations subscales [26].

Interestingly, weaker correlations were observed with the Oral Symptoms and Emotional Well-being domains. This pattern was similarly reported in a Brazilian study by Abanto et al., where social and functional limitations had a stronger association with caries than did emotional domains [27]. One explanation could be that children normalize oral pain or discomfort, or lack the vocabulary to articulate their emotional experiences. Younger children also tend to underreport symptoms due to limited self-awareness, which may contribute to this finding.

Additionally, DMFT and DT scores, reflecting caries in the permanent dentition, showed statistically significant but weaker correlations with total CPQ scores compared to primary dentition indices. This is expected, as many children in this age group are still in the mixed dentition phase. Nonetheless, the significant association between DT and CPQ scores highlights that even early caries in newly erupted permanent teeth can influence children’s self-perception and quality of life, underscoring the importance of initiating preventive measures at school entry. Although the observed correlation coefficients do not represent strong linear associations, they are meaningful in public health terms. Moderate correlations indicate that higher caries experience is consistently linked to poorer OHRQoL, particularly within the Social Well-being domain, reflecting the psychosocial burden of untreated caries. While modest at the individual level, these associations are important at the population level, identifying untreated caries as a key and modifiable determinant of child well-being. These findings justify early school-based oral health interventions and point to the need for larger longitudinal studies to validate effect sizes and inform targeted policies.

This study’s key strength lies in its focus on a tribal and underserved region, Rayagada district, where oral health data is scarce. The use of a validated, age-appropriate tool (CPQ₈₋₁₀) ensured reliability in capturing child-reported outcomes.

However, the study has certain limitations. Its cross-sectional design precludes establishing causal relationships. Moreover, self-reported data from younger children may be influenced by social desirability bias or limited comprehension. The complete absence of filled teeth also constrained the study’s ability to explore the effect of dental treatment on OHRQoL. In addition, the use of the CPQ₈₋₁₀ instrument, although widely validated in Western populations, has not been formally validated in the Indian context, which may influence the cultural applicability of responses.

Given the meaningful associations observed, future studies with larger, district-wide samples are warranted to validate these findings. Expanding the age range to include 6-12 years could capture the full transition from primary to permanent dentition. Longitudinal designs would allow assessment of how OHRQoL evolves with disease progression or treatment. Furthermore, integrating parent-reported CPQ scores, dietary recall, plaque indices, and clinical interventions could enhance the explanatory power of future studies. School-based oral health promotion programs and fluoride applications could be evaluated for their dual impact on clinical outcomes and quality of life.

In summary, this pilot study provides early evidence that dental caries significantly compromises OHRQoL in schoolchildren, particularly in terms of social functioning, whereas gingival health status did not show a significant association with OHRQoL in this sample. The results reinforce the need for early, accessible, and culturally appropriate preventive dental programs in tribal and rural regions of India.

## Conclusions

This pilot study conducted among 112 schoolchildren aged 8-10 years in Rayagada district revealed a burden of dental caries among 8-10-year-old schoolchildren, with significant age-wise differences in dt, dmft, and DT scores. and notable levels of OHRQoL concerns, particularly in the domains of Oral Symptoms and Social Well-being. Significant weak correlations were found between caries indices (especially dt and dmft) and CPQ scores, indicating that greater caries experience was associated with poorer perceived OHRQoL. However, no statistically significant associations were observed between gingival health status and CPQ domains. The findings highlight the need for targeted preventive oral health programs focusing on early caries detection, improved oral hygiene behaviors, and school-based interventions. However, given the pilot nature, the results should be interpreted with caution. Future research with larger and more diverse populations is warranted to validate these associations and to explore additional socio-behavioral and cultural factors influencing children’s oral health perceptions.

## Appendices

**Table 7:** Sociodemographic data collection form.

Section	Question	Response options
A. Demographics	Age (years)	☐ 8 ☐ 9 ☐ 10
	Gender	☐ Male ☐ Female ☐ Other
B. Oral Hygiene Practices	Type of cleaning tool used	☐ Finger ☐ Twigs ☐ Toothbrush
	Material used for cleaning teeth	☐ Herbal toothpaste ☐ Fluoridated toothpaste ☐ Toothpowder
	Method of cleaning	☐ Horizontal ☐ Vertical ☐ Circular
	Frequency of brushing	☐ Once per day ☐ Twice per day
